# Anti-Obesogenic Effects of Culinary Herbs Through Modulation of Inflammation and Metabolic Pathways

**DOI:** 10.3390/nu18060993

**Published:** 2026-03-20

**Authors:** Anna Winiarska, Agnieszka Tomczyk-Warunek, Karolina Jachimowicz-Rogowska, Małgorzata Kwiecień, Tomasz Czernecki, Magdalena Lis, Waldemar Kazimierczak

**Affiliations:** 1 Institute of Animal Nutrition and Bromatology, University of Life Sciences in Lublin, Akademicka St. 13, 20-950 Lublin, Poland; anna.mieczan@up.edu.pl (A.W.); malgorzata.kwiecien@up.edu.pl (M.K.); 2 Laboratory of Locomotor Systems Research, Department of Traumatology, Orthopedics and Rehabilitation, Medical University of Lublin, Jaczewskiego St. 8, 20-954 Lublin, Poland; agnieszka.tomczyk-warunek@umlub.edu.pl; 3 Department of Biotechnology, Microbiology and Human Nutrition, Faculty of Food Science and Biotechnology, University of Life Sciences in Lublin, Skromna St. 8, 20-704 Lublin, Poland; tomasz.czernecki@up.edu.pl; 4 Department of Biomedicine and Environmental Research, Faculty of Medicine, John Paul II Catholic University of Lublin, 20-708 Lublin, Poland; magdalena.lis@kul.pl (M.L.); waldemar.kazimierczak@kul.pl (W.K.)

**Keywords:** obesity, culinary herbs, metabolic processes, inflammation, microbiota

## Abstract

Obesity is considered a chronic disease that co-occurs with other disorders, including type 2 diabetes; therefore, the prevention and treatment of obesity are of utmost importance. The present review analysed the effects of bioactive compounds found in culinary herbs on the regulation of inflammatory processes through the modulation of inflammation and microbiota-dependent metabolic pathways. A total of 137 publications from 2010 to 2025 were reviewed. Few studies address the impact of culinary herbs on the gut microbiota in relation to obesity; however, analysing data on the effects of active compounds present in various herbs allows an assessment of their potential role in obesity prevention. This is a significant issue, as obesity is widespread, and the introduction of readily usable everyday food products may represent an important element of preventive strategies. Plant secondary metabolites, such as polyphenols, saponins, alkaloids, and flavonoids, exert strong antioxidant and anti-inflammatory activity, thus contributing to their beneficial effects on human health. Effective weight loss depends on the consistent maintenance of a healthy lifestyle, a requirement that can often be highly challenging. The daily use of herbs in meal preparation may reduce the risk of developing obesity or mitigate its severity. Herbs enhance the flavour of dishes and, additionally, help to reduce salt intake, thereby lowering the risk of cardiovascular disease, which is also an integral component of a healthy lifestyle.

## 1. Introduction

Obesity is a pathological condition characterised by abnormally high accumulation of adipose tissue leading to an increase in body mass significantly above the healthy levels defined for an individual’s age, sex, and ethnic background. It is considered a chronic malady associated with the emergence of other diseases, including type 2 diabetes; hence, the prevention and treatment of obesity is an important issue [1,2]. Under the current epidemiological circumstances, significant observations have been made in terms of the impact of obesity on the course of lung diseases [3]. Obesity is accompanied by low-intensity inflammation characterised by elevated activity of proinflammatory cytokines and adipokines, as well as release of interleukin-1β (IL-1β), interleukin-6 (IL-6), tumour necrosis factor-α (TNF-α), and leptin from the cells of white adipose tissue [4]. Multiple identified causes of obesity include factors that directly impact the organism’s internal processes: disorders of endocrine gland functions, elevated activity of proteins stimulating triglyceride synthesis, neuronal disorders, oxidative stress, metabolism disorders, and carbohydrate oxidation disorders [5,6]. The impact of these factors on the incidence of obesity can be minimised or at least limited through pharmacotherapy or diet therapy. However, the rather unsatisfactory results of pharmacological obesity treatment and its side effects [2] encourage the consideration of possible alternatives provided by phytotherapeutic medicines and nutraceuticals. The inclusion of natural substances in the treatment of diet-related conditions is considered safe due to the low concentrations of active compounds relative to the overall mass of the material as well as their high bioavailability. Moreover, in overweight patients or those only at risk of developing obesity, the use of natural substances may prove sufficient. In the context of obesity, phytotherapy shows particular promise, despite being underappreciated or even downright dismissed in general perception [7]. In the treatment of obesity, herbs should be considered second only to diet itself as a factor effectively preventing the excessive accumulation of adipose tissue. Culinary herbs deserve particular attention, as they can be widely incorporated into everyday life. Their additional advantage resides in the ability of herbs to enhance the sensory qualities of food while contributing to a reduction in dietary salt intake, which is currently widely acknowledged as one of the main contributors to the development of cardiovascular diseases [8].

The objective of this review was to analyse the viability of herbal supplements in diet therapy and prevention of obesity-related diseases. Based on data provided by global literature covering the past 15 years, the influence of herbs most frequently used in Poland for culinary purposes and active substances contained therein on the regulation of metabolic processes involved in body mass reduction was analysed.

## 2. Strategy for Information Retrieval from Available Sources

**Figure 1:**
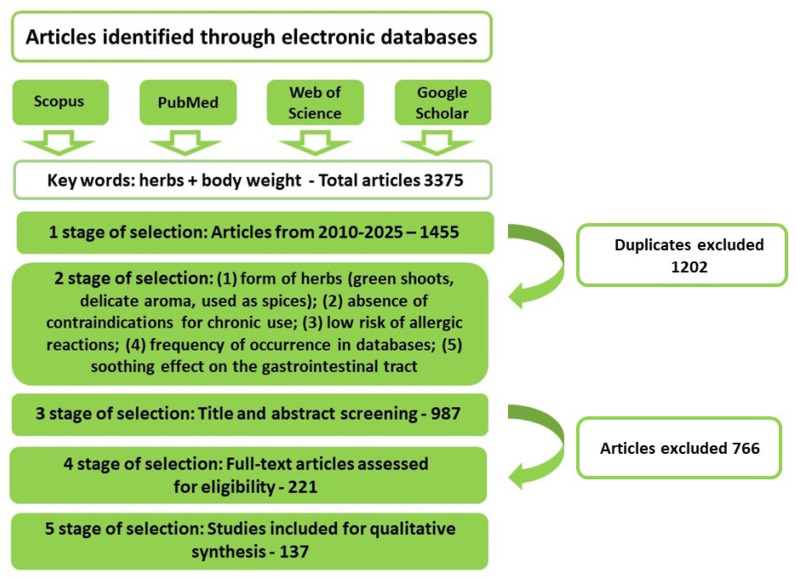
Methodological framework for reviewing available literature.

The literature review was conducted in June 2025 with the use of literature databases: Google Scholar, Scopus, Web of Science, and PubMed. In each searched database, the keywords “herbs” and “weight reduction” were queried in both Polish and English. An analysis of the titles and summaries of research and review publications revealed that the following herbs were most commonly referenced in the context of obesity: cress (*Lepidium sativum*), coriander (*Coriandrum* L.), sage (*Salvia officinalis* L.), and mint (*Mentha* sp.) were investigated most frequently to assess their role in obesity treatment. The search was restricted to papers released from 2010 to 2025. Additionally, the databases were searched for the individual or joint use of the following keywords: “herbs”, “weight loss”, “body mass reduction”, “obesity”, “metabolic processes”, “inflammation”, and “microbiota” (Figure 1). Papers that did not meet the subject-matter criteria were excluded, and the remaining articles were thoroughly reviewed in order to select the most relevant publications. The reference lists of the included papers were further examined to identify any valuable articles that may have been previously overlooked. Ultimately, a total of 137 publications were included in the analysis. Due to the narrative nature of this review, no formal meta-analytic procedure was employed; the results were synthesised qualitatively, emphasising the biological consistency of observed effects. PRISMA guidelines were used solely to systematise and facilitate the database review.

## 3. Etiopathology of Obesity

When an organism is in homeostasis, excessive energy is stored as triglycerides in the process of lipogenesis in the adipocyte cytoplasm. However, in the case of obesity, elevated levels of adipocytes are observed due to intensive preadipocyte differentiation [9]. Nonetheless, identification of potential adipogens susceptible to modulation by external factors may facilitate the applicability of adipogenesis regulation in controlling or even reversing obesity [9].

### 3.1. Oxidative Stress

**Figure 2:**
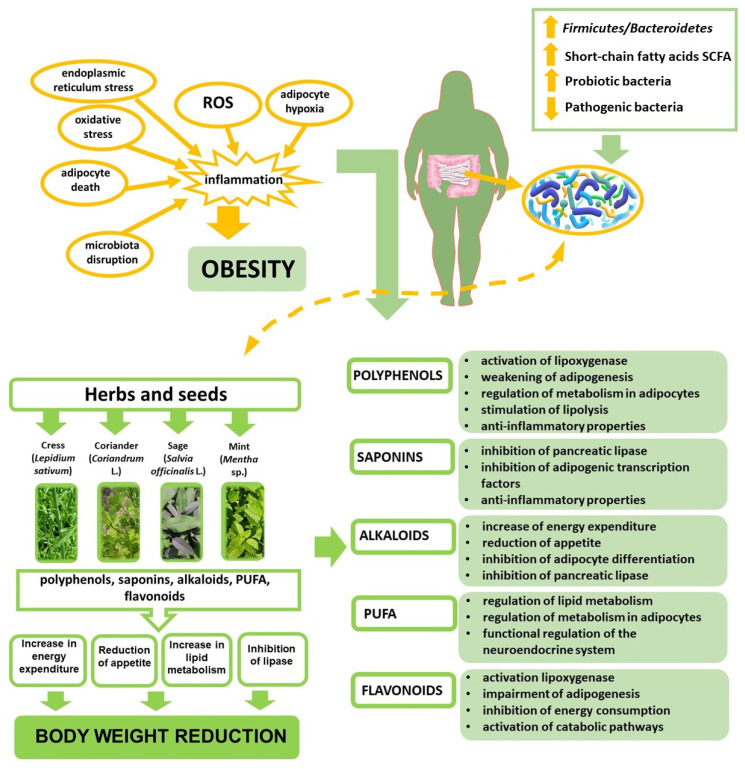
Bioactive substances in culinary herbs and their effect on the regulation of metabolic processes in obesity. ↑—increase; ↓—decrease.

The aetiology of obesity is multifaceted. A hallmark of this condition is systemic oxidative stress resulting from dysfunction of the antioxidative defence system involved in neutralisation of reactive oxygen species (ROS) and mitigation of chronic inflammation [4] (Figure 2). Oxygenation and incorrect protein structures contribute to dysfunction of adipocyte proteasomes, leading to hyperactivation of protein kinase and insulin resistance mediated by oxidative stress occurring in the hepatic endoplasmic reticulum and closely related to the emergence of inflammation [10,11,12]. ROS interact with polyunsaturated fatty acids in cellular membranes, thereby initiating the process of lipid peroxidation. This process results in protein modification, alterations in the membrane gradient, and consequently loss of integrity and irreversible cellular damage [12,13]. An increase in cellular ROS content may also stem from impairment of antioxidative mechanisms caused, above all, by a decrease in the cellular concentration of reduced glutathione (GSH) and the total pool of protein-bound -SH groups as well as changes in the activity of antioxidative enzymes [12]. Oxidative stress induces inflammatory reactions and triggers NF-κB (nuclear factor-κB) protein-dependent transcription of genes encoding various inflammatory mediators [14].

### 3.2. Hormones

One of the critical mechanisms underlying the emergence of obesity is believed to be related to disorders in lipid and carbohydrate homeostasis caused by genetic, environmental, and lifestyle factors [15]. Hormonal homeostasis is also important in the regulation of lipid and carbohydrate metabolism. In obesity-associated insulin resistance, the adipose tissue shows a reduced capacity to uptake glucose due to the reduced level of the type 4 glucose transporter (GLUT4), which is regulated primarily by insulin and due to elevated lipolysis and the consequent release of fatty acids and glycerol, which suppress the rate of lipogenesis [16].

### 3.3. Metabolism

Protein kinase activated by AMP (AMPK) integrates signals from hormonal and metabolic pathways to maintain the cell’s energy balance by, e.g., controlling lipid metabolism, through which it mediates the adaptation to dietary changes to preserve the homeostasis [2,17,18]. AMPK in skeletal muscles is activated by hormones, including leptin, adiponectin, and interleukin-6, whereas AMPK phosphorylation is inhibited by resistin [19]. Studies conducted on rats revealed that hepatic AMPK plays a significant role in the regulation of lipid metabolism between the liver and adipose tissue [20]. It was reported that, with the levels of glucose rising beyond the physiological norm, a sudden decrease in AMPK activity is observed, correlating with a decrease in threonine Thr172 phosphorylation in the α catalytic sub-unit [17]. The reduction in the AMPK activity detectable in diabetes and obesity may be the cause of inflammation, and chronic inflammation (concomitant with diminished antioxidative status) may be the cause of insulin resistance in obese patients [21]. It was additionally demonstrated that elevated TNF-α levels observed during inflammatory states may also trigger insulin resistance. TNF-α was reported to be overexpressed in the adipose tissue of obese humans and animals, while obese mice deprived of TNF-α or its receptor were found to be immune to insulin resistance problems [22]. In obese individuals, increased esterification and fatty acid uptake were observed, resulting in the accumulation of bioactive lipids in the tissues, e.g., ceramides, diacylglycerols, and acyl-CoA, which participate in the activation of proinflammatory kinases [18].

### 3.4. Molecular Mechanisms

Considerable scrutiny is currently also devoted to the molecular mechanisms regulating appetite and food intake, as well as the role played by appetite disorders in the aetiology of obesity [23]. Long-term exposure to the stressor may lead to a wide range of diseases, e.g., arterial hypertension or cardiac rhythm disorders, but can also trigger a decrease or increase in appetite, thus leading to either anorexia or obesity [24]. Under severe stress, the hypothalamic–pituitary–adrenal axis is activated. The hypothalamus secretes corticotropin-releasing hormone (CRH), which subsequently induces the release of adrenocorticotropic hormone (ACTH) [25]. ACTH stimulates the adrenal cortex to release corticosteroids, particularly cortisol which alters the metabolic rate for basic nutrients [25,26]. A study demonstrated the existence of common neural pathways mediating emotions (responsible for behavioural reactions to survival-related stimuli) and those regulating excessive consumption of high-calorie food [27].

### 3.5. Gut Microbiota

Gut bacteria can be categorised into several main groups: *Firmicutes*, *Bacteroidetes*, *Actinobacteria*, *Proteobacteria*, and *Verrucomicrobia*. Among these, *Firmicutes* and *Bacteroidetes* are predominant in adults, representing approximately 90% of all bacteria [28]. The gut microbiota regulates the development of metabolic disorders, including weight gain and fat accumulation in hepatic and adipose tissue [29]. It works by modulating appetite, affecting energy acquisition and absorption, and influencing gut barrier function. It also controls chronic inflammation, lipid metabolism, and glucose metabolism. The gut microbiota changes the secretion of hormones from enteroendocrine cells, including ghrelin, peptide YY, leptin, and GLP-1—hormones that regulate hunger and satiety [30,31]. This indicates that metabolites and components from gut microorganisms act as signals related to appetite. They help regulate hormone secretion and the immune system or directly affect hypothalamic neurons [32]. Numerous studies have reported an association between the composition of gut microbiota and obesity.

Research conducted in animals and humans has shown that individuals with excess body weight exhibit an imbalance primarily between the two dominant gut bacterial phyla: *Firmicutes* and *Bacteroidetes* [33,34]. Studies in rabbits have demonstrated that animals with high feed intake have a microbiota richer in *Bacteroides* and gamma-aminobutyric acid (GABA), and the gastric administration of this acid was effective in the stimulation of host feeding behaviour [35]. This suggests that GABA produced by the gut microbiota inhibits the secretion of satiety hormones, and differences in the composition of intestinal microbiota may lead to variations in food intake. In obese individuals, studies most frequently indicate a numerical dominance of *Firmicutes* over *Bacteroidetes*, leading to increased energy absorption from food and the occurrence of low-grade inflammation [29]. An obesity-promoting diet rich in fat and/or carbohydrates stimulates the proliferation of *Firmicutes*, *Prevotella*, and *Methanobrevibacter*, while reducing the abundance of short-chain fatty acid (SCFA)-producing bacteria, including *Bacteroides*, *Bifidobacterium*, *Lactobacillus*, and *Akkermansia* [36,37]. Reduced SCFA synthesis diminishes intestinal mucosal integrity and promotes the development of chronic inflammation [38]. Consequently, a compromised gut barrier allows bacterial metabolites to enter the blood circulation, potentially reducing insulin sensitivity and leading to impaired glucose metabolism and disrupted immune homeostasis [39]. The translocation of bacterial antigens across the gut wall contributes to subclinical inflammation, a critical factor in the initiation and maintenance of obesity [40]. It is believed that bacterial lipopolysaccharide, whose levels in the gut depend on diet, enhances the production of proinflammatory cytokines. Lipopolysaccharide is an endotoxin that forms part of the cell membrane of Gram-negative bacteria and cyanobacteria present in the gut lumen. An increase in circulating bacterial lipopolysaccharide in the body results in elevated glucose and triglyceride levels, increased inflammatory markers, and higher insulin resistance [40]. However, it is worth noting that some authors have not observed a relationship between excess body weight and an altered *Firmicutes*-to-*Bacteroidetes* ratio; some have even reported that the *Firmicutes*-to-*Bacteroidetes* ratio in individuals with obesity is lower than in lean subjects [41]. These findings suggest that inter-individual differences in intestinal microbiota sensitivity may largely determine the metabolic outcomes of excess body weight. The issue clearly warrants further investigation, as there is limited definitive information in this area. Understanding the mechanisms employed by the gut microbiota to modulate appetite and host metabolism may provide better insight into conditions associated with appetite dysregulation. Nevertheless, available studies suggest the potential of the gut microbiome in developing therapeutic interventions for management of obesity and its associated metabolic disorders.

## 4. Culinary Herbs Reducing Body Mass

Substances of plant origin are characterised by various biological properties, and some can directly influence certain causes of obesity; they also prevent a number of its accompanying diseases and show synergistic activity [42]. A number of studies conducted in both humans and laboratory animals have demonstrated the positive impact of herbs or herbal extracts on the reduction of body mass and stabilisation of blood parameters associated with obesity-related diseases (Table 1 and Table 2). Simultaneously, it is essential to recognise the dangers associated with the incorrect use of herbs, interactions between herbal active substances with various nutrients, and above all, the considerable variability in individual human responses to specific phytoingredients.

We recognise that laboratory animals are not perfect models for humans; nevertheless, they are commonly used as animal models in pharmacological and toxicological studies. Due to similar metabolic pathways, it is highly probable that results obtained in studies on laboratory animals can be largely extrapolated to humans, although complete certainty may not always be attainable. The available scientific literature presents few recent studies on the anti-obesogenic effects of culinary herbs in humans. This may be due to the fact that pharmacotherapy is more focused on obesity, while dietary interventions concentrate more on prevention and/or support of medical therapy. The results of clinical trials are presented in Table 2. They focused on only three of the herbs analysed in this review: *Coriandrum sativum* (n = 4), *S. officinalis* (n = 3), and *Mentha piperita* (n = 1). A total of 348 individuals participated in these studies, and the groups were small (6–67 individuals), which limits the interpretability of the results. In turn, studies on laboratory animals (n = 14) included 10–70 individuals. Laboratory animals, unlike humans, are characterised by a high degree of genetic similarity, which increases the reproducibility of results and, therefore, enhances the possibility of their reliable interpretation and the validity of the conclusions drawn.

### 4.1. Anti-Obesity Effect of Culinary Herbs—Research Review

As demonstrated in pharmacological studies, cress seeds (*Lepidium sativum*) show, e.g., antidiabetic, antioxidative, gastrointestinal, anti-inflammatory, cardiovascular, and hypolipidaemic activity. Such conditions tend to accompany excessive body mass, and normalisation of biochemical blood parameters is likely to improve the general health of affected individuals. In Turkish traditional medicine, cress was used to improve digestion [65]. In an experiment conducted on rats suffering from diabetes, a hypoglycaemic effect of water or methanolic extracts of cress seeds was observed [45,66]. It was reported that the rats also showed stabilisation of general cholesterol levels and blood serum triglycerides. The positive effects of cress seeds in terms of the analysed parameters were associated with the presence of alkaloids. Administration of pulverised cress seeds in obese rats contributed to a significant reduction in the serum levels of apelin (a bioactive peptide from the adipokine family), glucose, insulin, insulin resistance, total cholesterol, triglycerides, LDL-C, VLDL, and MDA. It also significantly increased the levels of GSH and HDL-C [43]. As reported by El-Dakak et al. [44], administration of a water extract of cress seeds to diabetic rats normalised their body mass, which may be attributable to its anti-hyperglycaemic effects. Hyperinsulinaemia is closely correlated with the level of apelin; moreover, apelin expression increases in response to hyperinsulinaemia in obese rats and humans [67,68]. Cress seeds contain 0.4% alkaloids, 0.42% flavonoids, approx. 3% saponins, and 0.6% tannins—all of which show strong antioxidative properties [65]. Oxidative stress reflects an imbalance between the organism’s antioxidative capacity and the accumulation of toxic oxidative products, consequently leading to tissue damage. It is believed that, in humans, oxidative stress is considered a factor involved in the emergence of obesity-related complications [43]. Seeds and other parts of cress plants are commonly used to facilitate body mass normalisation in Mediterranean countries given their content of amylase and pancreatic lipase inhibitors [2]. Studies conducted on rats have demonstrated that garden cress seeds may alleviate metabolic and oxidative disturbances induced by a high-fat diet through a reduction in apelin activity and lowering VLDL and triglyceride levels [43]. Apelin is an adipocytokine—a hormone secreted by adipose tissue cells—whose elevated levels have been observed in obesity-related conditions [69]. It is found in various tissues and organs, e.g., in the gastrointestinal tract, adipose tissue, brain, pancreas, blood, and the circulatory system [43]. Its significant role in the pathogenesis of obesity and regulation of metabolism has been confirmed; it acts through the hypothalamic thirst centre to suppress appetite, modulate the secretion of pituitary and hypothalamic hormones, and enhance insulin sensitivity [69].

Coriander (*C. sativum* L.) seeds are rich in essential oils, whose hypoglycaemic and hypolipidaemic effects were confirmed in obese and diabetic patients [57,70]. The cited studies also demonstrated that water extracts of coriander seeds administered orally to obese rats with hyperglycaemia and hyperlipidaemia attenuated metabolic syndrome and reduced atherosclerotic outcomes in the animals while enhancing their cardioprotective effects. The hypoglycaemic and hypolipidaemic effects of coriander seeds have been thoroughly studied and documented in studies conducted on obese animals with hyperlipidaemia [70,71]. In turn, diabetics receiving pulverised coriander seeds for 6 weeks were observed to show clearly reduced indicators of metabolic syndrome and atherosclerosis as well as an increase in cardioprotective indices [57]. In a study involving diabetic rats, administration of coriander seeds resulted in a decrease in liver mass and visceral fat. Excessive visceral fat accumulation and increased liver mass are regarded as indicators of the onset of obesity and metabolic syndrome [72]. Accumulation of fat in the viscera is related to the production of inflammation mediators. Proinflammatory cytokines produced during the expansion of adipose tissue impact the energy balance in diseases triggered by excessive fat accumulation, e.g., obesity and diabetes [73]. A study conducted on rats revealed that the incorporation of coriander seeds in the animals’ feed rations reduced the accumulation of hepatic and visceral fat due to the presence of active substances influencing the metabolism of lipids in the liver and visceral tissues [46]. Studies conducted on rats also demonstrated that, while the addition of coriander seeds did not alter the hepatic lipid content, it modified the profile of fatty acids in visceral fat by significantly increasing the content of monounsaturated and polyunsaturated acids [46]. A particularly important observation pertains to the improved omega-3/omega-6 acid ratio, which may indeed account for the health benefits of consuming coriander. Its seeds contain between 10 and 30% of oil, which in turn contains approx. 20–30% of polyunsaturated fatty acids, 15–20% of which is linoleic acid [65]. Human studies have also confirmed the effect of coriander seeds on blood parameters in obesity (Table 2). Diabetics taking pulverised coriander seeds for 6 weeks were observed to show significantly reduced indicators of metabolic syndrome and atherosclerosis, as well as an increase in cardioprotective indices [57]. In individuals with osteoarthritis, the use of coriander leaf powder (5 g/day) for 60 days resulted in improved antioxidant parameters in blood [58]. In healthy individuals and diabetes patients, a reduction in lipid parameters and blood glucose levels was observed [59,60].

Although the exact mechanisms of the anti-obesogenic effects of substances present in sage (*S. officinalis* L.) remain unknown, numerous studies have demonstrated the contribution of sage infusions or extracts to lowering the body mass and improving atherogenic parameters. A sage infusion included in the diet for obese rats reduced the animals’ body mass and visceral fat mass, and lowered the levels of triglycerides, total cholesterol, LDL, and C-reactive protein in blood serum [53]. Sá et al. [61] demonstrated that, after a two-week intake of a water-based extract of sage (300 mL, twice daily), obese patients showed improvements in their lipid profiles—reduced LDL and total cholesterol levels and increased HDL levels. In a study on rats suffering from polycystic ovary syndrome, the animals received a sage infusion, which reduced their blood glucose levels and atherogenic factors and improved their antioxidative parameters [56]. In a group of mice with induced obesity receiving a methanol-based sage extract for 5 weeks, blood glucose, triglycerides, and plasma insulin level were reduced. Additionally, mitigation of inflammation (increased levels of anti-inflammatory cytokines IL-2, IL-4, and IL-10 and reduced levels of proinflammatory cytokines IL-12, TNF-α, and KC/GRO) in the plasma and inhibition of lipogenesis in adipocytes were found [54]. The anti-inflammatory effects of sage extracts were also demonstrated in a study on isolated human cells of mature subcutaneous adipocytes [74]. As reported by El-Sayed et al. [55], administration of sage to obese rats resulted in a reduction in body mass and the levels of total cholesterol, triglycerides, LDL, glucose, ALT, and AST as well as an increase in the HDL level. Thanks to the presence of carnosic acid, carnosol, roylenoic acid, 7-metoxyrosmanol, and oleanolic acid, a methanolic extract of *S. officinalis* leaves significantly inhibited the activity of pancreatic lipase, stabilised the serum level of triglycerides, and reduced body mass gain and visceral fat accumulation in mice receiving a high-fat diet [15,54]. Mice suffering from hyperlipidaemia receiving sage essential oil (4 mg/kg b.m.) showed a decrease in their body mass gain, lipid indices, functional liver and kidney disorders, and ROS production [74]. Slightly fewer studies are available on humans, but their results are similar to those obtained in laboratory animals (Table 2). Sage infusions are as effective as metformin, an oral antidiabetic drug used in the management of type 2 diabetes, which suggests their influence on gluconeogenesis at the hepatic level (high capacity to capture glucose, reduce gluconeogenesis in response to glucagon, and increase insulin susceptibility) [75]. Studies conducted in a group of people with hyperlipidaemia [62] and hyperlipidaemia coexisting with type 2 diabetes [63] also showed a positive effect of sage extracts on lipid parameters (total cholesterol, triglycerides, LDL-C, VLDL-C, HDL-C) in blood. The anti-inflammatory properties of sage extracts were also demonstrated in a study on isolated human cells of mature subcutaneous adipocytes [76]. Sage contains polyphenols, including flavonoids, as well as saponins and phytosterols [53]. Carnosic, rosmarinic, and caffeic acids present in sage contribute considerably to the antioxidative protection of the organism [75]. In turn, carnosic acid, carnosol, roylenoic acid, 7-metoxyrosmanol, and oleanolic acid exhibit inhibitory activity against pancreatic lipase [15].

Various mint cultivars (fresh and dried) are widely used in the traditional medicine of many countries, for example to support body mass reduction, and for their antioxidative, anti-inflammatory, antidiabetic, and cardioprotective effects. Mint can also alleviate digestive discomfort, e.g., flatulence, stomachache, and indigestion [77,78]. A number of mechanisms have been proposed in the literature to elucidate mint’s potential to contribute to weight loss. Its effects include suppression of pancreatic lipase activity, stimulation of lipolysis and lipid metabolism, stimulation of thermogenesis, limitation of adipocyte differentiation, reduction of oxidative stress and inflammation, inhibition of cholesterol and triglyceride synthesis, and reduction of appetite [79]. Active compounds with anti-obesogenic properties include flavonoids (catechin, epicatechin, myricetin, apigenin, naringenin, luteolin, kaempferol, rutin, and quercetin) as well as phenolic acids (rosmarinic, caffeic, gallic, chlorogenic) [79]. Peppermint (*M. piperita* L.) enhances digestive processes, particularly fat digestion, by increasing the release of stomach juices. Thanks to its content of menthol and carvacrol, mint can block calcium channels, thereby allowing stomach smooth muscles to relax [80,81]. Studies conducted with the use of bovine pancreatic lipase demonstrated an inhibitive effect of mint essential oil, which the authors attributed mainly to the presence of carvone (depending on the mint cultivar, it can constitute even up to approx. 80% of all oil ingredients) as well as other monoterpenes [77]. A positive impact of *Mentha longifolia* L. oil and powder was reported in the context of oxidative stress, glucose tolerance, and insulin resistance in Wistar rats with diet-induced obesity [82]. Thirty-day administration of peppermint juice (*M. piperita* L.) resulted in a reduction of glycaemia in 41.5% of the subjects, total cholesterol and transaminases in approx. 70%, triglycerides in nearly 60%, and the LDL fraction in over 50% [64]. Moreover, the cited paper reported an increase in the HDL fraction as well as lowered blood pressure and BMI in approximately 50% of the patients. Twenty-one-day oral peppermint juice administration triggered a statistically significant reduction in glucose levels in rats with alloxan-induced diabetes, which was attributed by the authors to the content of polyphenols and antioxidative properties of mint [51]. After the administration of *M. piperita* essential oil to rats with induced diabetes, researchers observed alleviation of diabetes-related anaemia, an increase in leukocyte and blood platelet counts, reduction of blood glucose levels, an increase in serum concentrations of insulin and C-peptide, and improvement of the antioxidative status [49]. In the cited study, a histological analysis of the liver and pancreas evidenced tissue regeneration and reduction of the extent of degenerative lesions, while immunohistochemical tests revealed increased expression of Bcl-2 (anti-apoptotic protein) and insulin. Similarly, in an experiment where rats with induced diabetes received a water extract of *Mentha spicata* leaves, the levels of glucose, total cholesterol, triglycerides, LDL, and MDA fractions were reduced [50]. A study on pregnant Wistar rats revealed that the administration of mint tea (*M. spicata* labiatae) resulted in a body mass reduction relative to animals receiving pure water [83]. At the same time, the authors also observed lowered body mass of the offspring, which indicated potential risks of the treatment in pregnant women.

### 4.2. Anti-Obesity Effect of Active Substances Contained in Culinary Herbs Through Modulation of Metabolic Processes

Various culinary herbs contain a wide range of bioactive substances capable of regulating metabolic processes (Figure 2). They can support body mass reduction through multiple mechanisms, including (1) increasing energy expenditure by reducing metabolic rates; (2) suppressing appetite via stimulation of noradrenalin synthesis and activation of the sympathetic nervous system, which triggers the sense of satiation; (3) enhancing lipid metabolism, e.g., through β-adrenergic receptor activation, which initiates lipolysis in white adipocytes—lipolysis is activated by stimulating the adrenergic receptor; and (4) inhibiting pancreatic lipase production by covalent bonds with serine at the enzyme active site [5,6,9]. Active substances promoting body mass loss include polyphenol, polyunsaturated fatty acids (PUFA), saponins, alkaloids, and oligosaccharides [84]. Properties responsible for inhibiting lipase are exhibited by polyphenols, flavonoids, saponins, alkaloids, and terpenoids [15].

It has been demonstrated that flavonoids, in particular flavonols, activate lipoxygenase and suppress adipogenesis through modulation of the AMP-activated protein kinase pathway; its activation is induced by elevated AMP concentrations during energy deficiency periods, which promotes catabolic pathways and inhibition of energy consumption processes [18,84]. Flavonoids most probably modulate the number of cellular signal pathways regulating digestion of carbohydrates, accumulation of fat, insulin release rates, and glucose uptake in insulin-sensitive tissues [84]. A number of investigations have found that polyphenols modulate metabolism in adipocytes to suppress the fatty tissue expansion and reduce their vitality and differentiation [6,84]. Phenolic, chlorogenic, and coumaric acids have been reported to effectively inhibit the proliferation of preadipocyte cells in a murine model [6,85]. They also reduce the biosynthesis of fatty acids and accumulation of triglycerides, promote lipolysis and β-oxidation of fatty acids, and mitigate inflammation via downregulation of the expression of strong proinflammatory TNFα adipokines, monocyte-1 chemotactic proteins (MCP-1), and type-1 plasminogen activator inhibitor (PAI-1), while enhancing the production of anti-inflammatory adiponectin in adipocytes [86,87,88]. Polyphenols are known to modulate signalling pathways, such as protein kinase activated by AMP—AMPK (which plays a key role in energy homeostasis; deactivation of AMPK is associated with type 2 diabetes, diet-induced obesity, insulin resistance, and other metabolic diseases), the receptor activated by γ peroxisome proliferators, and NF-κB—regulating adipogenesis, antioxidative and anti-inflammatory response, and the expression of certain adipokine genes reacting to oxidation, including TNF-α [4,6]. Saponins show anti-inflammatory, anti-lipidaemic (through inhibition of pancreatic lipase), hypocholesterolaemic, and hypoglycaemic properties [2]. Studies on rats demonstrated that saponins increase AMPK phosphorylation in a dose-dependent manner, which suggests their direct modulatory role in AMPK activation in adipocytes [89]. Saponins also attenuate the accumulation of internal fat via suppression of adipogenic transcription factors involved in AMPK signalling, i.e., CCAAT/α transcription factors (C/EBPα) and γ2 peroxisome proliferator-activated receptors (PPARγ2) [2]. Flavonoids are proposed to modulate multiple cellular signalling pathways, thereby affecting carbohydrate digestion, lipid storage, insulin secretion dynamics, and glucose uptake in insulin-responsive tissues [5]. Alkaloids enhance energy expenditure, suppress appetite, and inhibit adipocyte differentiation and pancreatic lipase secretion. While the majority of alkaloids function as α-adrenergic antagonists, some show the properties of β-adrenergic antagonists [5]. Meanwhile, the effects of PUFAs entail maintenance of the balance between energy uptake and expenditure, regulation of lipid metabolism, support of adequate metabolism in adipocytes, and functional regulation of the neuro-endocrine system [90].

**Figure 3:**
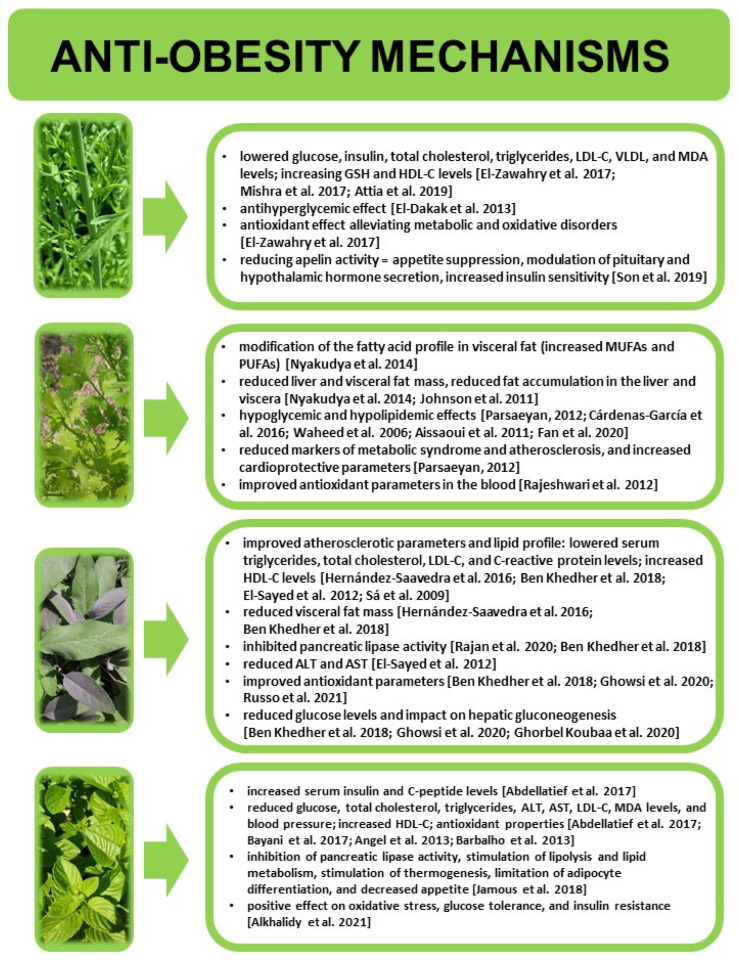
Comparison of the anti-obesity mechanisms of the four herbs: cress (*Lepidium sativum*), coriander (*Coriandrum* L.), sage (*S. officinalis* L.), and mint (*Mentha* sp.) [15,41,43,45,46,49,50,51,53,54,55,56,57,58,59,60,61,64,66,69,70,71,72,74,75,79,82].

Figure 3 shows comparison of the anti-obesity mechanisms of the four herbs: cress (*Lepidium sativum*), coriander (*Coriandrum* L.), sage (*S. officinalis* L.), and mint (*Mentha* sp.).

### 4.3. Anti-Obesogenic Effects of Bioactive Compounds from Herbs via Modulation of Intestinal Microbiota

The interaction between herbal consumption and the intestinal microbiota arises from two factors: (1) the effects of bioactive herbal compounds on the composition of intestinal microbiota, and (2) the metabolic responses of these compounds in the human organism mediated through the gut microbiota [91]. In individuals with excess body weight, an elevated *Firmicutes*-to-*Bacteroides* ratio is often observed [36,37]. *Firmicutes* utilize long-chain carbohydrates, thereby producing more nutrients, which may lead to increased energy intake and subsequent weight gain [92]. The intestinal microbiota in overweight individuals efficiently extracts energy from dietary intake. Certain bioactive compounds present in herbs are able to alter the ratio of these bacteria. A study conducted in healthy human volunteers demonstrated that frequent consumption of polyphenol-rich herbs was associated with an increased abundance of *Firmicutes*, but was negatively correlated with *Proteobacteria* abundance [28]. Culinary herbs contain a range of bioactive compounds that influence the gut microbiota composition, shifting it in an anti-obesogenic direction. These include polyphenols, saponins, alkaloids, polyunsaturated fatty acids (PUFAs), and flavonoids.

**Figure 4:**
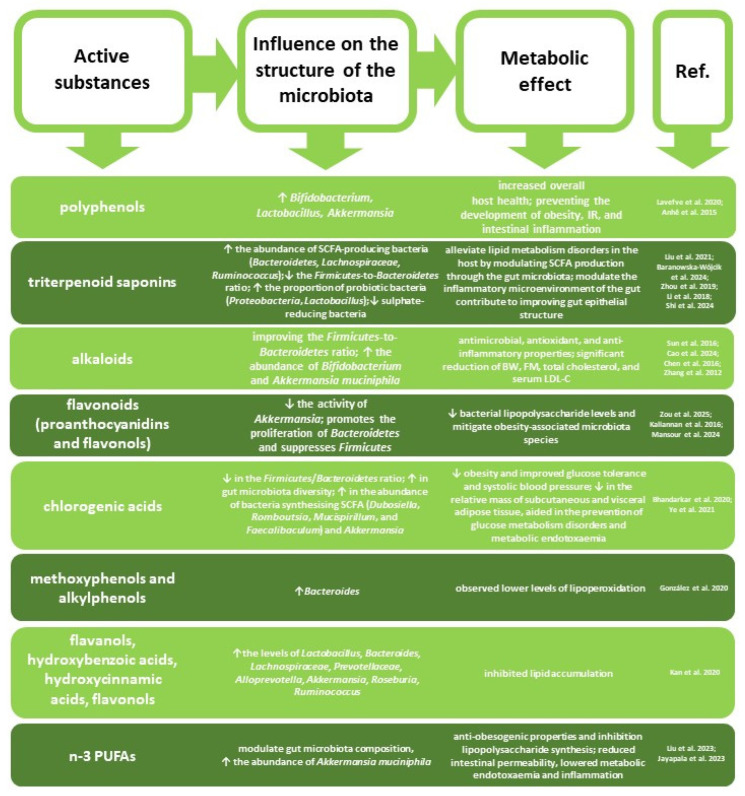
Influence of herbal active ingredients on metabolism via modulation of gut microbiota. ↑—increase; ↓—decrease [84,93,98,99,100,101,102,103,104,105,107,109,110,114,115,116,124,125,126,127].

The interaction between polyphenols and the gut microbiota occurs during the digestion of dietary components. Upon reaching the colon, polyphenols are transformed by the gut microbiota into low-molecular-weight bioactive metabolites, which affect microbiome diversity, including the *Firmicutes*-to-*Bacteroidetes* ratio [92]. A prebiotic effect of polyphenols has been observed, stimulating the growth of probiotic bacteria such as *Bifidobacterium*, *Lactobacillus*, and *Akkermansia*, thereby contributing to increased SCFA concentrations and overall host health [93]. Glycosidic bond cleavage in polyphenols produces glycans, which serve as a key nutrient source for the intestinal microbiota, particularly for *Bacteroidetes* [94]. The prebiotic properties of polyphenols are attributed to their metabolites and involve: (1) stimulation of bacterial growth and metabolism by disrupting pathogenic microbial cell membrane function, (2) regulation of tight junction protein expression, and (3) regulation of the balance in the synthesis of pro- and anti-inflammatory T lymphocytes [95,96]. This has a direct impact on the gut microbiota composition by stimulation of the growth of probiotic strains and inhibition of pathogenic bacteria. Furthermore, the activation of the Nrf2 (nuclear factor erythroid 2-related factor 2) signalling pathway via PI3K/Akt (phosphoinositide-3-kinase/Akt) confers protection to IPEC-J2 cells (intestinal porcine enterocyte cell line) against oxidative stress, thereby mitigating gut barrier disruption, as evidenced in a study on resveratrol [97]. Studies in C57BL/6J mice with diabetes induced by high-fat and high-sugar diet revealed a protective effect of polyphenols, which increased the abundance of *Akkermansia* spp., thus preventing the development of obesity, insulin resistance, and intestinal inflammation [98]. On the other hand, the microbiota supports the absorption of polyphenols in the gastrointestinal tract, as they exhibit low bioavailability in the human small intestine [99]. Bacteria associated with polyphenol metabolism primarily include *Flavonifractor plautii*, *Slackia equolifaciens*, *Slackia isoflavoniconvertens*, *Adlercreutzia equolifaciens*, *Eubacterium ramulus*, and *Eggerthella lenta*, as well as *Lactobacillus* and *Bifidobacterium* spp., with different bacteria exhibiting affinity for different groups of polyphenols [100]. In the distal intestine, polyphenols are hydrolysed and metabolised by intestinal enzymes and the gut microbiota (Figure 4). They are then transported to the liver via the portal circulation, where they undergo further transformations. Absorption of metabolites is possible primarily due to glucuronidation and sulphation, leading to the formation of sulphate, glucuronide, and methyl conjugates [100]. Without the participation of microbiota, effective absorption and utilisation of polyphenols would not be possible.

Studies involving laboratory animals have also shown that saponins can influence the gut microbiota composition by raising the abundance of SCFA-producing bacteria, e.g., *Bacteroidetes*, *Lachnospiraceae*, and *Ruminococcus*, reducing the *Firmicutes*-to-*Bacteroidetes* ratio, and enhancing the proportion of probiotic bacteria, including *Proteobacteria* and *Lactobacillus* [99,100,101,102]. Consequently, saponins may alleviate lipid metabolism disorders in the host by modulating SCFA production through the gut microbiota [103]. Herbal triterpenoid saponins have been reported to strengthen beneficial bacteria, reduce sulphate-reducing bacteria, and alter the inflammatory microenvironment in the murine gut [104]. They also contribute to improving gut epithelial structure by increasing the expression of anti-inflammatory cytokine IL-4 and reducing the expression of pro-inflammatory cytokines TNF-α, IL-1β, and IL-18 [104].

Studies on animals have demonstrated that alkaloids isolated from various plants can positively modulate gut microbiota disrupted under different stress conditions by improving the *Firmicutes*-to-*Bacteroidetes* ratio, enhancing intestinal mucosal integrity through their antimicrobial, antioxidant, and anti-inflammatory effects, and promoting mucin production [84,105,106]. Bactericidal properties of alkaloids have also been reported [107]. The administration of alkaloids isolated from Ramulus Mori led to a favourable shift in the *Firmicutes*-to-*Bacteroides* ratio at the phylum level and increased the abundance of *Bifidobacterium* and *Akkermansia muciniphila* at the genus level, significantly reducing body weight, fat mass, and the levels of total cholesterol and serum low-density lipoprotein in obese mice [108].

Omega-3 polyunsaturated fatty acids (PUFAs) can alter the diversity and abundance of gut microbiota, which, in turn, can influence PUFA metabolism and absorption [109]. PUFAs are associated with anti-obesogenic effects, as demonstrated in mice receiving a high-calorie diet [110]. Dietary intake of n-3 PUFAs has been shown to modulate the gut microbiota composition, increasing the abundance of *Akkermansia muciniphila*, which is considered anti-obesogenic and inhibits lipopolysaccharide synthesis. Elevated levels of omega-3 PUFAs enhance intestinal alkaline phosphatase production and secretion, which induces changes in the gut bacterial composition that reduce lipopolysaccharide production and intestinal permeability, thereby lowering metabolic endotoxaemia and inflammation, as demonstrated in mice studies [111]. Studies on mice fed a high-fat diet have shown that supplementation with 10-hydroxy-cis-12-octadecenoic acid (HYA), an initial gut microbiota metabolite of linoleic acid, attenuates obesity without inducing adipose tissue inflammation caused by arachidonic acid; instead, it improves metabolic status via free fatty acid receptors [112]. Conversely, research indicates that a high omega-6/omega-3 PUFA ratio diminishes the beneficial effects of omega-3 on gut microbial diversity and abundance, whereas a balanced ratio promotes a healthier microbiota profile [113]. Moreover, an inappropriate omega-3/omega-6 ratio leads to a significant increase in the *Firmicutes*-to-*Bacteroidetes* ratio, ultimately contributing to excess body weight and obesity [109]. This is particularly important given the excess of omega-6 and deficiency of omega-3 in modern Western diets. Studies on Fat-1 mice have confirmed the protective effects of omega-3 PUFAs against antibiotic-induced gut dysbiosis and obesity [114].

Flavonoid metabolism promotes the proliferation of *Bacteroidetes* and suppresses *Firmicutes* [115]. Flavonoids are degraded into glycans, and glycan-degrading enzymes are more abundant in *Bacteroidetes* than in *Firmicutes*, allowing *Bacteroidetes* to survive and proliferate following human consumption of flavonoids. Moreover, flavonoids, particularly proanthocyanidins and flavonols, reduce the activity of *Akkermansia*, a mucin-degrading bacterium known to decrease bacterial lipopolysaccharide levels and mitigate obesity-associated microbiota species [116,117]. An in vitro study employing human faecal samples demonstrated that flavonoids positively influence the growth of probiotic *Bifidobacterium* and *Lactobacillus* strains [118].

Active substances contained in herbs exert prebiotic effects, which are reflected in their influence on the intestinal microbiota structure. SCFAs produced by commensal intestinal bacteria exhibit anti-inflammatory effects, modulate the composition of the intestinal microbiota, and help maintain human health by influencing intestinal integrity [119]. A lack of permeability in the intestinal barrier leads to the entry of bacterial metabolites into the circulatory system, which can impair insulin sensitivity, glucose metabolism, and immune homeostasis and may lead to excess body weight [120]. SCFAs are products of bacterial metabolism of dietary fibre occurring in the gastrointestinal tract. Elevated SCFA concentrations lower intestinal pH, inhibiting the growth of *Enterobacteriaceae*, which produce lipopolysaccharides [100,121]. The most important SCFAs are butyrate, propionate, and acetate. These acids play a role in body weight control through various mechanisms: (1) stimulation of satiety hormones and regulation of appetite suppression (acetate and propionate) and (2) inhibition of lipolysis and improvement of insulin sensitivity (acetate and butyrate) [122,123].

## 5. Potential Restrictions on the Use of Culinary Herbs

The use of culinary herbs is generally considered safe. However, contraindications may occur in certain chronic diseases due to possible interactions with active drug ingredients, as well as during pregnancy and breastfeeding. Allergic reactions are also possible.

In pharmacotherapy, a key aspect is the potential change in the properties of the drug’s active substance (antagonistic or synergistic effects), its potency (weakening or strengthening), and the duration of action (shortened or prolonged) [128]. Interactions occurring in the pharmacodynamic phase (which may occur during the absorption, distribution, and excretion of the drug) can affect the concentration of the drug’s active ingredients in blood [128]. The active substances of some herbs act by modulating the activity of the cytochrome P450 (CYP) enzyme and monoamine oxidase (MAO) in the first stage of drug biotransformation [129]. Such interactions can lead to health complications in patients. Importantly, the amount of herbs consumed plays a significant role in these interactions, although toxic effects may occur even after consuming relatively small amounts [129]. Several herbal components have been identified as natural inhibitors of P450 enzymes: terpenoids, phenylpropanoids, flavonoids, alkaloids, and quinones [130]. The safety of culinary herbs has not been frequently assessed in high-quality clinical trials conducted among pregnant and breastfeeding women. Available results most often originate from research on herbal medicines, with a focus on their popularity [131,132]. Caution should be exercised during pregnancy and lactation, as many active ingredients from herbs pass into breast milk, while knowledge of the use of herbal remedies to support breastfeeding and the postpartum period is passed down through generations in many cultures [133,134]. Research indicates that some herbal plants should be excluded from the diet of pregnant women, but these are not commonly used culinary herbs. These include *Abrus precatorius*, *Achyranthes aspera*, *Ailanthus excelsa*, *Aloe vera*, *Ginkgo biloba*, *Moringa oleifera*, *Ricinus communis*, *Ruta graveolens*, and *Valeriana officinalis* [135]. As with drug interactions, the type of herbal spice used is crucial during pregnancy and lactation, as the amount consumed is small and serves solely as a meal addition.

In the case of allergies to culinary herb ingredients, both IgE-dependent and IgE-independent mechanisms are particularly important, with common cross-reactivity to pollen (birch and mugwort) [136]. Mustard, celery, coriander, fennel, cumin, anise, and pepper are most frequently mentioned in the context of allergies [136]. However, diagnosis remains difficult due to the limited number of standard tests, which is why oral food challenge is the gold standard.

The available literature indicates that it is impossible to quantify the levels of culinary herbs that may be hazardous to humans. Rather, attention should be paid to the plant species, which is essential for assessing and minimising clinical risk. Culinary herbs are typically used as spices; therefore, very small amounts are generally consumed. Some culinary herbs can also be used as teas (in our case, mint or sage) or dietary supplements, but in such cases, they should not be considered culinary herbs, i.e., food additives, but rather stand-alone food products, whose content of active substances interacting with drugs may be higher [137]. This is likely the reason why the available studies on contraindications to herbal use primarily concern dietary supplements.

## 6. Summary and Perspectives

Both the effects and mechanisms of the contribution of herbs to the treatment or prevention of obesity remain controversial. Nonetheless, clinical studies have shown their viability in the struggle against obesity, while experiments on animals have elucidated some of the mechanisms of their anti-obesogenic effects, or at least suggested potential explanations thereof. As follows from clinical reports, herbs cause few adverse effects and generally show acceptable levels of safety. Particular attention should be paid to the molecular mechanisms related to the active substances present in herbs. Research findings indicate that culinary herbs may mitigate obesity and metabolic syndrome by reducing oxidative stress and chronic inflammation and by improving the gut microbiome and the metabolic profile of gut bacteria. However, it should always be acknowledged that human organisms may differ significantly in their individual responses to individual phytonutrients.

Culinary herbs may have anti-obesogenic, anti-diabetic, and anti-hyperlipidaemic properties. Being readily available, they can be used to support overweight individuals in managing excess weight and to facilitate the maintenance of a healthy body weight. Effective weight loss depends on the ability to sustain a healthy lifestyle, which can often be highly challenging. Daily incorporation of herbs into meal preparation may reduce the risk of developing obesity or mitigate its severity. Herbs enhance the flavour of dishes and, additionally, help to reduce salt intake, thereby lowering the risk of cardiovascular diseases [8], which is also an important component of a healthy lifestyle.

## Figures and Tables

**Table 1:** Effects of herbs on body weight reduction and blood serum parameters—a review of animal studies.

Herbs	Protective Effect	Design	Animals	References
*Lepidium sativum*	↓ body weight; ↓ adiposity index; ↓ serum triglycerides; ↓ plasma total cholesterol; ↑ serum HDL-C; ↓ serum LDL-C; ↓ serum VLDL-C; ↓ apelin; ↓ glucose; ↓ serum insulin; ↓ insulin resistance index; ↓ serum MDA; ↑ serum GSH	*L. sativum* seeds powder was administered orally with a high-fat diet at 6 g/kg/day for 8 weeks	Wistar and Sprague Dawley male rats fed a high-fat diet	[43]
*Lepidium sativum*	↓ glucose; ↓ serum triglycerides; ↓ plasma total cholesterol; ↓ urea; ↓ uric acid; ↓ creatinine; ↓ serum AST; ↓ serum ALT; ↓ serum ALP; ↑ insulin; ↓ serum MDA; ↑ plasma total antioxidant potential	Aqueous extract of *L. sativum* seeds was administered via a stomach tube at 50 mg/kg b.w. for 60 days	Streptozotocin-induced diabetic male albino rats	[44]
*Lepidium sativum*	↓ glucose; ↓ creatinine; ↓ serum ALP; ↓ plasma total cholesterol; ↓ serum MDA	Aqueous extract of *L. sativum* seeds was administered orally at 20 mg/kg b.w. for 16 days	Streptozotocin-induced diabetic rats	[45]
*Coriandrum sativum*	↑ omega 3: omega 6 fatty acid ratio in visceral adipose tissue	Crushed coriander seeds (500 mg/kg b.w.) were administered with chow for 5 weeks	Female Sprague–Dawley rats	[46]
*Coriandrum sativum*	↓ fasting blood glucose; ↓ body weight; ↓ serum AST; ↓ serum ALT; ↓ urea; ↓ creatinine; ↓ plasma total cholesterol; ↓ serum triglycerides; ↓ serum LDL-C; ↑ serum HDL-C	A polyphenol fraction of *C. sativum* seeds (25 or 50 mg/kg b.w.) was administered orally for 28 days	Alloxan-induced diabetic Swiss albino mice	[47]
*Coriandrum sativum*	↓ serum glucose; ↑ activity of the beta cells; ↑ insulin	*C. sativum* ethanolic extract was administered at 200 and 250 mg/kg	18 male Wistar streptozotocin-induced diabetic rats	[48]
*Mentha piperita*	↓ blood glucose; ↑ serum insulin ↑ C-peptide; ↑ antioxidant status	Diabetic rats received different doses of peppermint essential oil—40 and 80 mg/kg or a hypoglycaemic agent	70 adult albino rats with diabetes	[49]
*Mentha spicata*	↓ fasting blood sugar; ↓ total cholesterol; ↓ triglycerides; ↓ LDL-C; ↓ serum malondialdehyde	*M. spicata* extract was administered orally at 300 mg/kg to diabetic rats for 21 days	24 Wistar albino rats	[50]
*Mentha piperita*	↓ blood glucose level	Peppermint juice was administered orally at 0.29 g/kg once a day for 21 days	30 adult male and female alloxan-induced diabetic Wistar rats	[51]
*Mentha piperita*	↓ glucose; ↓ cholesterol; ↓ LDL-C; ↓ triglycerides; ↑ HDL-C	The animals received *M. piperita* juice at 0.29 g/kg once a day (at early morning) for 30 consecutive days	20 offspring from diabetic Wistar rats	[52]
*Salvia officinalis*	↓ body weight; ↓ abdominal fat mass; ↓ serum triglycerides; ↓ total cholesterol; ↓ LDL-C; ↓ CRP	Infusions of *S. officinalis* were freshly prepared (1% *w*/*v*) and administered *ad libitum* for 6 weeks	40 male Sprague–Dawley rats	[53]
*Salvia officinalis*	↓ blood glucose; ↓ plasma insulin levels; ↓ oral glucose tolerance test; ↓ HOMA-IR; ↓ triglycerides; ↓ non-esterified fatty acids; ↓ IL-12; ↓ TNF-α; ↓ KC/GRO; ↑ insulin sensitivity; ↑ IL-2; ↑ IL-4; ↑ IL-10	Animals were treated with sage methanolic extract (100 and 400 mg kg−1/day bid), or rosiglitazone (3 mg kg−1/day bid) for 5 weeks	32 male diet-induced obese mice	[54]
*Salvia officinalis*	↓ body weight; ↓ serum triglycerides; ↓ serum cholesterol; ↓ LDL-C; ↓ VLDL-C; ↓ glucose; ↓ AST; ↓ ALT; ↑ HDL-C	Animals received a high-fat diet supplemented with 2%/4% of sage or purslane or their mixture for 28 days	48 male albino rats on a high-fat diet with induced obesity	[55]
*Salvia officinalis*	↓ serum HDL-C; ↓ glucose; ↓ total cholesterol; ↓ LDL-C; ↓ atherogenic index levels; ↑ serum TAC	Sage tea was administered in place of water *ad libitum* for 14 days	18 female Wistar rats with induced PCOS	[56]

↓—decreased or inhibited concentration or activity relative to the untreated group; ↑—increased concentration or activity relative to the untreated group; AST—aspartate aminotransferase; ALT—alanine aminotransferase; LDL-C—low-density lipoproteins; VLDL-C—very low-density lipoproteins; HDL-C—high-density lipoprotein; GSH—reduced glutathione; TAC—antioxidant total capacity; ALP—alkaline phosphatase; TNF-α—tumour necrosis factor α; CRP—C-reactive protein; IL-2, IL-4, IL-10, IL-12—interleukins; KC/GRO—keratinocyte-derived chemoattractant/human growth-regulated oncogene; MDA—malondialdehyde; HOMA-IR—Homeostatic Model Assessment—Insulin Resistance.

**Table 2:** Effects of herbs on blood serum parameters—a review of clinical studies.

Herbs	Protective Effect	Design	Population	References
*Coriandrum sativum*	↓ glucose; ↓ plasma total cholesterol; ↓ serum triglycerides; ↓ serum LDL-C; ↓ atherosclerotic index; ↑ cardioprotective indices	Patients consumed 2 capsules of coriander seed powder per day for 6 weeks	50 patients with type 2 diabetes mellitus	[57]
*Coriandrum sativum*	↓ lipid peroxidation in erythrocytes and plasma; ↑ vit. C and beta-carotene; ↓ uric acid; ↓ ceruloplasmin; ↑ GSH; ↑ glutathione-S-transferase; ↓ urea; ↓ creatinine	Oral administration of coriander leaf powder (5 g/day) for 60 days	Osteoarthritis patients	[58]
*Coriandrum sativum*	↓ glucose; ↓ cholesterol; ↑ TAC	Volunteers received *C. sativum* seed powder for 4 months	120 volunteers from Mexico	[59]
*Coriandrum sativum*	↓ glucose	Oral administration of dried powder, aqueous and alcoholic extracts of the plant at low (2.5 g tid) and high (4.5 g tid) doses 3 times daily for 14 days	20 patients with type 2 diabetes mellitus (10 taking oral hypoglycaemic agents)	[60]
*Salvia officinalis*	↓ LDL-C; ↓ total cholesterol; ↑ HDL-C; ↑ SOD; ↑ CAT	Subjects received 300 mL of sage tea taken twice daily for 4 weeks	6 healthy female volunteers aged 40–50 years	[61]
*Salvia officinalis*	↓ total cholesterol; ↓ triglycerides; ↓ LDL-C; ↓ VLDL-C; ↑ HDL-C	Subjects received sage leaf extract (one 500 mg capsule every 8 h for 2 months)	67 hyperlipidaemic patients aged 56.4 ± 30.3 years old	[62]
*Salvia officinalis*	↓ fasting glucose; ↓ HbA1c; ↓ total cholesterol; ↓ triglycerides; ↓ LDL-C; ↑ HDL-C	Subjects received sage leaf extract (one 500 mg capsule for 3 months)	40 hyperlipidaemic type 2 diabetic patients aged 40–60 years old	[63]
*Mentha piperita*	↓ glycaemia; ↓ BMI; ↓ total cholesterol levels; ↓ triacylglycerides; ↓ LDL-C; ↓ GOT levels; ↓ GPT levels; ↓ SBP; ↑ HDL-C	Subjects received peppermint juice twice daily (2 × 200 mL) at a concentration of 20 g of peppermint leaves/200 mL of water for 30 days	25 students between 18 and 45 years old	[64]

↓—decreased or inhibited concentration or activity relative to the untreated group; ↑—increased concentration or activity relative to the untreated group; BMI—body mass index; LDL-C—low-density lipoproteins; VLDL-C—very low-density lipoproteins; HDL-C—high-density lipoprotein; SBP—systolic blood pressure; GOT—glutamic-oxaloacetic transaminase; GPT—glutamic-pyruvic transaminase; SOD—superoxide dismutase; CAT—catalase; GSH—reduced glutathione; TAC—antioxidant total capacity; HbA1c—glycated haemoglobin.

## Data Availability

No new data were created or analysed in this study.

## Abbreviations

The following abbreviations are used in this manuscript:

SARS-CoV-2	severe acute respiratory syndrome coronavirus 2
IL-1β, IL-2, IL-4, IL-6, IL-10, IL-12, IL-18	interleukins
TNF- α	tumour necrosis factor-α
ROS	reactive oxygen species
GSH	reduced glutathione
NF-κB	nuclear factor-κB
GLUT-4	type 4 glucose transporter
AMPK	phospho-AMP-activated protein kinase
CRH	corticotropin-releasing hormone
ACTH	adrenocorticotropic hormone
GLP-1	glucagon-like peptide-1
GABA	gamma-aminobutyric acid
SCFA	short-chain fatty acid
LDL-C	low-density lipoproteins
VLDL-C	very low-density lipoproteins
MDA	malondialdehyde
HDL-C	high-density lipoprotein
KC/GRO	keratinocyte-derived chemoattractant/human growth-regulated oncogene
ALT	alanine aminotransferase
AST	aspartate aminotransferase
BMI	body mass index
Bcl-2	anti-apoptotic protein
PUFA	polyunsaturated fatty acids
MCP-1	monocyte-1 chemotactic proteins
PAI-1	type 1 plasminogen activator inhibitor
C/EBPα	CCAAT-enhancer-binding proteins α
PPARγ2	γ2 peroxisome proliferator-activated receptors
Nrf2	nuclear factor erythroid 2-related factor 2
PI3K/Akt	phosphoinositide-3-kinase/Akt
IPEC-J2	intestinal porcine enterocyte cell line
HYA	10-hydroxy-cis-12-octadecenoic acid
